# The Comparison of Regional RNFL and Fundus Vasculature by OCTA in Chinese Myopia Population

**DOI:** 10.1155/2018/3490962

**Published:** 2018-01-31

**Authors:** Yuanjun Li, Hamza Miara, Pingbo Ouyang, Bing Jiang

**Affiliations:** ^1^Department of Ophthalmology, Hunan Clinical Research Center of Ophthalmic Disease, The Second Xiangya Hospital, Central South University, Changsha, Hunan, China; ^2^Department of Ophthalmology, Guangdong Provincial Key Laboratory of Malignant Tumor Epigenetics and Gene Regulation, The Sun Yat-sen Memorial Hospital, Sun Yat-sen University, Guangzhou, Guangdong, China

## Abstract

**Purpose:**

To determine the correlations between peripapillary vessel density, retinal nerve fibre layer (RNFL) thickness, and myopic indices at retina quadrants with optical coherence tomography angiography (OCTA) in Chinese.

**Methods:**

Fifty-six subjects with a mean spherical equivalent (MSE) of −3.63 ± 0.29 D were included. Peripapillary RNFL thickness and retinal vessel density in four sectors (superior, nasal, inferior, and temporal quadrants) were determined by OCTA, and correlations of the main outcomes were analyzed.

**Results:**

Negative correlations were found between the peripapillary RNFL thickness and axial length (AL) at superior (*r* = −0.335, *P* = 0.001) and inferior (*r* = −0.551, *P* < 0.001) quadrants. There was a significant positive correlation with spherical equivalent (SE) at the corresponding quadrants as well as at the nasal quadrant (*r* = 0.339, *P* = 0.001; *r* = 0.379, *P* < 0.001; and *r* = 0.209, *P* = 0.039, resp.). Peripapillary retinal vessel density was also negatively correlated with AL at the nasal quadrant (*r* = −0.392, *P* < 0.001), and only at the nasal quadrant, there was a positive correlation between the peripapillary vessel density and SE (*r* = 0.319, *P* = 0.001).

**Conclusions:**

The degree of myopia and elongation of AL were negatively correlated with peripapillary RNFL thickness at superior and inferior quadrants and with peripapillary retinal vessel density at the nasal quadrant.

## 1. Introduction

Worldwide, myopia is among the most common ocular disorder with high prevalence in Asian population [1]. As a major cause of low vision and legal blindness, myopia in a greater stage may lead to macular complications, including posterior staphyloma, retinoschisis, lacquer crack formation, chorioretinal atrophy, and myopic choroidal neovascularization [2].

Optical coherence tomography angiography (OCTA) is a novel noninvasive technology that provides fast, depth-resolved visualization of the retinal and choroidal microvasculature [3–6]. Previously, an increasing number of OCTA studies have focused on pathological eye changes in myopia, including the association between retinal vasculature, retinal nerve fibre layer (RNFL), and eye structural parameters. By quantitatively assessing the microvasculature of the retina and choriocapillaris in the myopic eyes, OCTA studies have illustrated a reduced retinal capillary microvasculature and increased flow deficit in choriocapillaris in the eyes with greater myopia [5]. Besides the reported decreased peripapillary retinal perfusion in the highly and pathological myopic eyes as compared to the emmetropic eyes, it was found that the retinal vasculature was positively correlated with RNFL thickness in myopic subjects [1, 7].

However, it remains unknown whether there are significant sectional correlations between regional RNFL thickness, retinal vessel distribution, and eye structural parameters, since the previous OCTA studies about myopia only assessed the association between vascular and RNFL structure in an overall way. Because different eye diseases such as myopia and glaucoma often originate and exaggerate regionally with different patterns [8], it is worthwhile to explore and test the RNFL and retinal vasculature from locational perspectives. Furthermore, there are sparse researches about the specific regional correlations between RNFL, vasculature, and myopic parameters though the course of myopia development. Bearing this in mind, we aimed to investigate the global and regional correlations between RNFL thickness, vessel density, and eye structural parameters via OCTA assessment, in various stages of myopia.

## 2. Methods

### 2.1. Participants

This cross-sectional, observational study was conducted in the Department of Ophthalmology, the Second Xiangya Hospital, Central South University. The study population consisted of healthy and myopia subjects recruited from October 2016 to January 2017. This study was conducted in accordance with the tenets of the Declaration of Helsinki (1964) and fully approved by the ethics committee of the Second Xiangya Hospital, Central South University. Informed consent as to the scientific objectives and process of the study was obtained from each subject.

### 2.2. Inclusion and Exclusion Criteria

Inclusion criteria were age of 18 years or more and presence of myopia in the studied eyes, as assessed by the refraction error and axial length. For each participant, one or both eyes meeting the criteria were included in the study. Participants had no known eye diseases as determined by full ophthalmic examinations.

The studied eyes were assigned to one of the four groups according to refraction: emmetropia (EM; mean spherical equivalent (MSE) 0.50 D to −0.50 D), mild myopia (MIM; MSE −0.75 D to −2.75 D), moderate myopia (MOM; MSE −3.00 D to −5.75 D), and high myopia (HM; MSE ≤ −6.00 D).

Exclusion criteria were any history of prior vitreous or retinal surgery or evidence of retinal diseases (other than myopic degeneration, such as age-related macular degeneration, macular hole, and foveal hypoplasia) affecting the retinal or choroidal vasculature by history or examination, presence of media opacities preventing reliable retinal thickness which prevents good visualization of the retinal structure, having systemic diseases such as glaucoma or diabetes mellitus which might affect the ocular circulation, and medication usage within 2 weeks of measurements.

### 2.3. Data Collection and Examinations

All participants in the study underwent comprehensive ophthalmologic examination including spherical equivalent (SE) refraction measurement with an autorefractometer (KR-8800, Topcon, Tokyo, Japan), slit-lamp biomicroscopy, and fundus examination. An IOL Master (Carl Zeiss Inc., Jena, Germany) keratometer was used for measuring anterior chamber depth, *K* values, and axis length. OCTA (Optovue RTVue XR Avanti, Optovue Inc., Fremont, California, USA) was used for retinal nerve fibre layer (RNFL) and vascularization assessment. Demographic data (age, gender), general medical history, and ophthalmologic history were also collected.

### 2.4. Optical Coherence Tomography Angiography

OCTA images were acquired with AngioVue (Optovue RTVue XR Avanti, Optovue Inc., Fremont, CA, USA) using automated segmentation algorithms. The system has an A-scan rate of 70 kHz scans per second, with a light source centered on 840 nm and a bandwidth of 45 nm. A 6 mm × 6 mm (36 mm^2^) area OCTA acquisition centered on the optic disc was performed, to record the overall and regional retinal RNFL thickness and vessel density. Three-dimensional (3D) OCTA scans were acquired by using two repeated B-scans at 304 raster positions, with each B-scan consisting of 304 A-scans. With a B-scan frame rate of 210 frames per second, each OCTA volume scan can be acquired in approximately 3 s. Two volumetric raster scans, including one horizontal priority (x-fast) and one vertical priority (y-fast), were obtained consecutively. The volumetric scans were processed by the split-spectrum amplitude-decorrelation angiography (SSADA) algorithm. All scans were reviewed by an examiner to ensure correct imaging and sufficient scan quality. Signal strength index (SSI) <45 and severe artifacts (double vessel pattern, loss of fixation, motion artifacts, and/or segmentation error resulting in poor outlining of vascular networks) in scans were the OCTA exclusion criteria. All of this processing can be achieved using the contained software (Optovue Inc., software V.2014.2.0.65).

**Figure 1:**
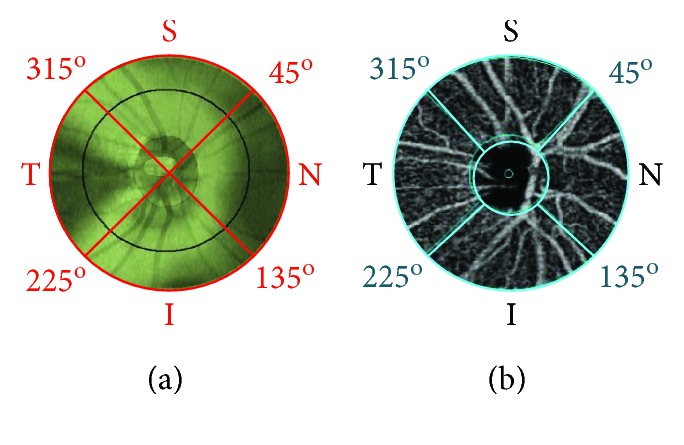
Peripapillary RNFL thickness and vessel density at quadrants were identified by OCTA. The boundaries of segmentation are indicated by the red lines ((a) peripapillary RNFL thickness) or blue lines ((b) peripapillary vessel density). Peripapillary quadrants are identified as 6.0 mm radial scans 90° apart, around the central point of the optic disc. S, superior, radial scan from 315° to 45°; N, nasal, from 45° to 135°; I, inferior, from 135° to 225°; T, temporal, from 225° to 315°.

Peripapillary retinal vessel density was imaged by OCTA with lateral fixation position of the subjects and quantified with the Angio Retina program. Measurements were automatically performed at four peripapillary quadrants: superior (S), inferior (I), nasal (N), and temporal (T) (Figure 1).

### 2.5. Statistical Analysis

Raw data results were processed by statistical analysis software (IBM SPSS version 22.0 for Mac, SPSS Inc., Chicago, IL, USA). The Spearman rank correlation analysis was performed to determine the relationships between the overall and regional RNFL thickness, vessel density, and myopia-related eye structural parameters. Qualitative variables are presented as numbers and percentages. Quantitative variables are presented as means and standard deviations. Nonparametric ANOVA comparing the 4 tested eye groups used the nonparametric Kruskal-Wallis test. Qualitative variable (gender) was compared by the Fisher exact test. *P* < 0.05 was considered statistically significant.

## 3. Results

### 3.1. Demographics

A total of 56 healthy subjects (98 eyes) were included in this cross-sectional OCTA study. Demographic characteristics and eye structural measurements are shown in Table 1. Of the examined eyes, 17 eyes in the subjects had emmetropia. Twenty-seven eyes had mild myopia, 33 had moderate myopia, and 21 had high myopia. There were 42 eyes from men and 56 eyes from women; there were 48 right eyes (OD) and 50 left eyes (OS). The mean age of all subjects was 24.5 ± 5.1 years. Between the studied groups, statistically significant differences were found in terms of MSE (*P* < 0.001), axial length (AL) (*P* < 0.001), average peripapillary RNFL thickness (*P* < 0.001), and peripapillary vessel density (*P* = 0.023). Otherwise, there were no significant differences in age, laterality, or gender between the four groups (all *P* > 0.05).

Generally, significant differences among the four groups were found at the superior and inferior quadrants of the RNFL thickness in the peripapillary area according to our OCTA results. At the inferior quadrant, the peripapillary RNFL thickness decreased as the myopia grew. The measurement was 139.4 ± 10.4 *μ*m, 140.3 ± 14.1 *μ*m, 132.1 ± 11.3 *μ*m, and 123.1 ± 14.9 *μ*m, in the emmetropia, mild myopia, moderate myopia, and high myopia groups, respectively (*P* < 0.001). Similarly, at the superior quadrant, the greater myopic eyes had thinner RNFL thickness of 137.5 ± 10.7 *μ*m, 140.0 ± 11.2 *μ*m, 131.9 ± 13.9 *μ*m, and 124.2 ± 14.8 *μ*m, in the emmetropia, mild myopia, moderate myopia, and high myopia groups, respectively (*P* = 0.002), although the superior RNFL in mild myopia was slightly thicker than that in emmetropia. However, no statistically significant change was found in the nasal or temporal RNFL thickness (*P* = 0.211 and *P* = 0.919, resp.) among the four groups.

Overall, the highly myopic eyes had a lower peripapillary vessel density, as shown in Table 1. There were significant differences in the peripapillary vessel density between the emmetropia and the myopic groups at the superior quadrant (*P* = 0.028), the nasal quadrant (*P* = 0.016), and the temporal (*P* = 0.015) quadrant. The inferior quadrant was the only one where peripapillary vessel density did not differ significantly between the four MSE groups.

### 3.2. Correlation between Regional Peripapillary RNFL Thickness and Myopia

**Figure 2:**
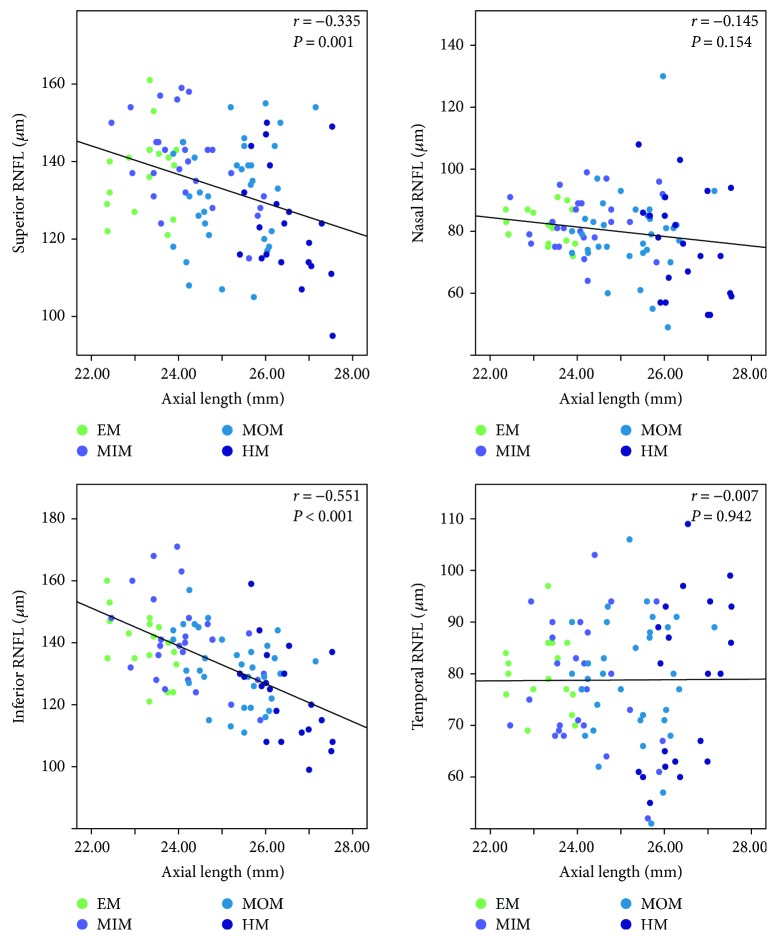
Scatterplots illustrating the linear (black line) associations between axial length (mm) and OCTA peripapillary quadrant (superior, nasal, inferior, and temporal) RNFL thickness (*μ*m) measurement of the studied eyes. *r*, correlation coefficient from the Spearman rank correlation analysis; EM, emmetropia, MSE 0.50 D to −0.50 D; MIM, mild myopia, MSE −0.75 D to −2.75 D; MOM, moderate myopia, MSE −3.00 D to −5.75 D; HM, high myopia, MSE ≤ −6.00 D.

**Figure 3:**
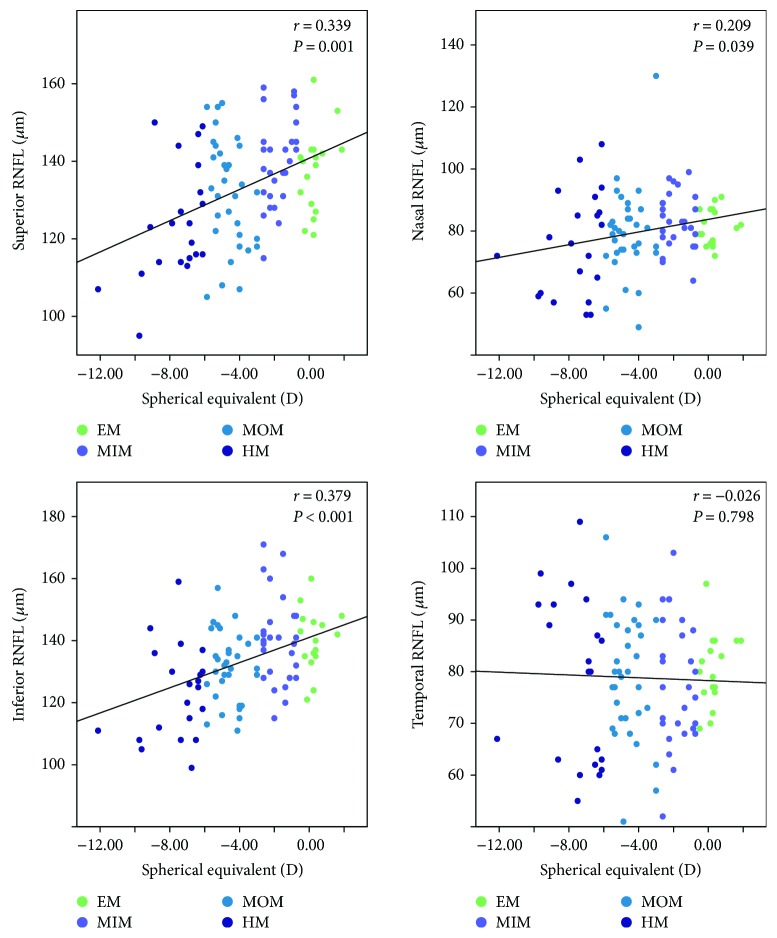
Scatterplots illustrating the linear (black line) associations between spherical equivalent (D) and optical coherence tomography angiography (OCTA) peripapillary quadrant (superior, nasal, inferior, and temporal) RNFL thickness (*μ*m) measurement of the studied eyes. *r*, correlation coefficient from the Spearman rank correlation analysis; EM, emmetropia, MSE 0.50 D to −0.50 D; MIM, mild myopia, MSE −0.75 D to −2.75 D; MOM, moderate myopia, MSE −3.00 D to −5.75 D; HM, high myopia, MSE ≤ −6.00 D.

The results of the Spearman correlation analysis of the RNFL thickness at quadrants, AL, and SE are shown in Table 2. Generally, there were significantly negative correlations between peripapillary RNFL thickness and AL as shown in the table (*r* = −0.450, *P* < 0.001; Table 2 and Figure 2). Among the quadrants, there were significant negative correlations at superior and inferior regions (*r* = −0.335, *P* = 0.001 and *r* = −0.551, *P* < 0.001, resp.; Table 2 and Figure 2). At the nasal and temporal quadrants, however, no statistical significant association was found between the peripapillary RNFL measurements and AL (*r* = −0.145, *P* = 0.154 and *r* = −0.007, *P* = 0.942, resp.; Table 2 and Figure 2). As expected, there was a significant positive correlation with SE at the corresponding quadrants as well as at the nasal quadrant (*r* = 0.339, *P* = 0.001; *r* = 0.379, *P* < 0.001; and *r* = 0.209, *P* = 0.039, resp.; Table 2 and Figure 3), which agreed with the trend between peripapillary RNFL thickness and AL. At the temporal quadrant, the peripapillary RNFL thickness did not correlate with the SE (*r* = −0.026, *P* = 0.798; Table 2 and Figure 3). Collectively, these results indicate that peripapillary RNFL thickness thins as the myopia degree increases, especially at the superior and inferior quadrants of the retina.

### 3.3. Correlation between Regional Peripapillary Vessel Density and Myopia

**Figure 4:**
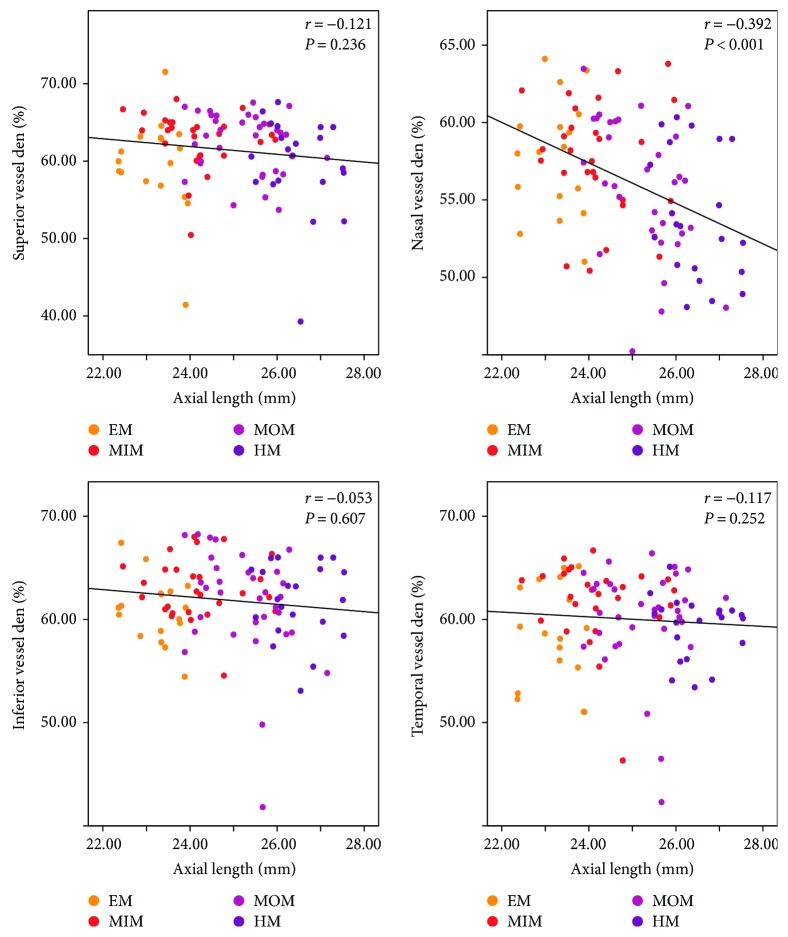
Scatterplots illustrating the linear (black line) associations between axial length (mm) and optical coherence tomography angiography (OCTA) peripapillary quadrant (superior, nasal, inferior, and temporal) vessel density (%) measurement of the studied eyes. *r*, correlation coefficient from the Spearman rank correlation analysis; EM, emmetropia, MSE 0.50 D to −0.50 D; MIM, mild myopia, MSE −0.75 D to −2.75 D; MOM, moderate myopia, MSE −3.00 D to −5.75 D; HM, high myopia, MSE ≤ −6.00 D.

**Figure 5:**
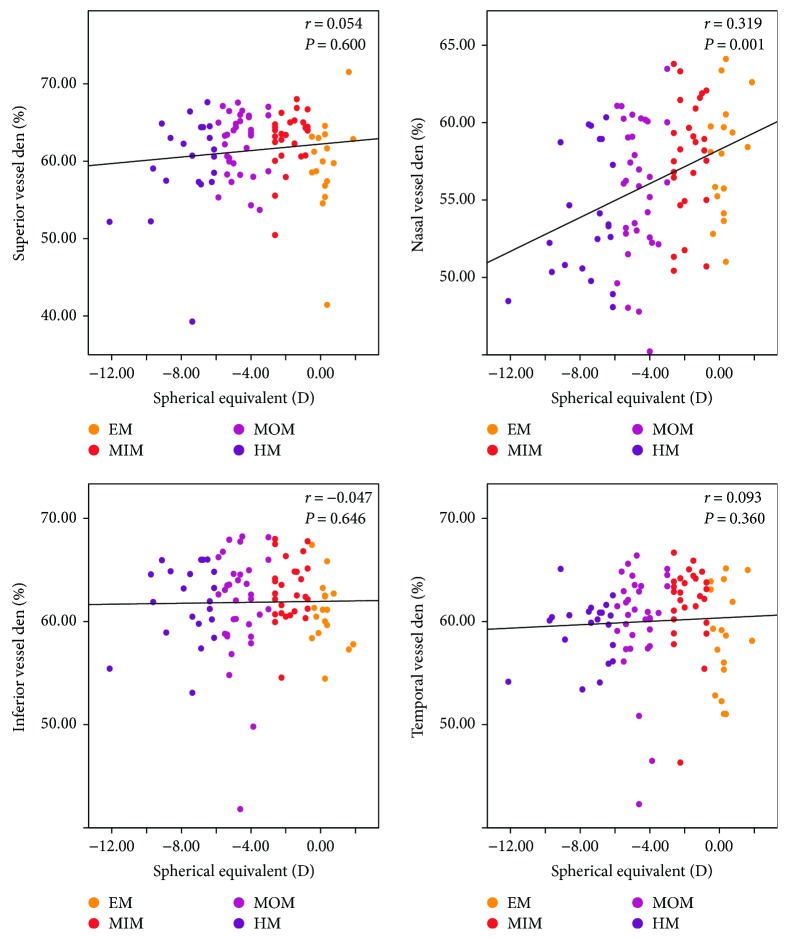
Scatterplots illustrating the linear (black line) associations between spherical equivalent (D) and optical coherence tomography angiography (OCTA) peripapillary quadrant (superior, nasal, inferior, and temporal) vessel density (%) measurement of the studied eyes. *r*, correlation coefficient from the Spearman rank correlation analysis; EM, emmetropia, MSE 0.50 D to −0.50 D; MIM, mild myopia, MSE −0.75 D to −2.75 D; MOM, moderate myopia, MSE −3.00 D to −5.75 D; HM, high myopia, MSE ≤ −6.00 D.

Generally, there were significantly negative correlations between peripapillary vessel density and AL (*r* = −0.234, *P* = 0.020; Table 3). However, as for the four quadrants, only at the nasal quadrant, there was a statistically inverse correlation between peripapillary vessel density and AL (*r* = −0.392, *P* < 0.001; Table 3 and Figure 4). At the superior, inferior, and temporal quadrants, peripapillary vessel density did not correlate with AL (*r* = −0.121, *P* = 0.236; *r* = −0.053, *P* = 0.607; and *r* = −0.117, *P* = 0.252, resp.; Table 3 and Figure 4). Unlike the peripapillary RNFL thickness, no statistical correlation was found between the average peripapillary vessel density and SE (*r* = 0.152, *P* = 0.135; Table 3). As for the four regions, similarly only at the nasal quadrant, there was a positive correlation between the peripapillary vessel density and SE (*r* = 0.319, *P* = 0.001; Table 3 and Figure 5). The correlation efficiency was not statistically significant at the superior, inferior, and temporal quadrants (*r* = 0.054, *P* = 0.600; *r* = −0.047, *P* = 0.646; and *r* = 0.093, *P* = 0.360, resp.; Table 3 and Figure 5). Overall, these results suggest that the peripapillary vessel density reduces mainly at the nasal quadrant as the myopia increases, indicating a graphical pattern different from that of peripapillary RNFL thickness.

## 4. Discussion

In the present study, the graphical relationship between peripapillary RNFL and vasculatures with the stages of myopia has been quantified with the OCTA technique. It was shown that as myopia increases, the reduction of peripapillary RNFL thickness mainly occurred at the superior and inferior quadrants of the retina. Characterization of RNFL thickness in the myopic eyes with OCT-based techniques has been described in the literature (Table 4). Our results are in agreement with those of the previous OCT studies that reported a significantly lower RNFL thickness in high myopia at 12, 1, and 7 o'clock regions (12 o'clock sectors) or superior/inferior areas (quadrant sectorial division), as compared to the mild to moderate myopia [9, 10]. Recently, using Cirrus HD-OCT (software version 6.0) and three-dimensional analysis, Seo et al. reported that RNFL thickness of the 1, 2, 5, 6, and 12 o'clock sectors was significantly thinner in moderate to high myopia than in mild myopia (*P* < 0.001) [11]. Interestingly, their results suggest thicker RNFL measurements at the 8, 9, and 10 o'clock sectors in moderate and high myopia as compared with the mild myopia (*P* = 0.001, 0.003, and <0.001, resp.) [11]. With OCT (version 3, Stratus OCT, Carl Zeiss Medites Inc.), Leung et al. studied 115 healthy control and 115 myopic eyes (MSE −7.31 ± 3.04 D) and showed that there were significant negative correlations between RNFL thickness and AL and negative SE [9]. Using OCTA (RTVue-XR OCT, Optovue Inc., software V.2014.2.0.65), Wang et al. also showed that highly myopic eyes had longer AL and thinner RNFL thickness [1]. Although there were studies using OCT to quantify the RNFL with different methods of retinal division, results from the present study first made use of the OCTA technique to describe the graphical details and correlations of regional peripapillary RNFL thickness and AL at different myopia degrees. It may indicate that the OCTA could be used as an effective and efficient way to study the RNFL structure and vascular network in the myopic eyes, which is a promising alternative of the OCT.

On the other hand, the study showed that only at the nasal quadrant, there was a statistically negative correlation between the peripapillary vessel density and AL in the studied groups, indicating that the elongation of the globe affected nasal retinal vasculature significantly. A wide range of previous studies has already shown a decreased peripapillary perfusion in the greater myopic eyes. Wang and colleagues in their vascular-related OCTA research showed a lower peripapillary retinal perfusion measurement, including retinal flow index and vessel density, with respect to emmetropic eyes in Chinese population [1]. In another study with the OCTA technique, the same research team demonstrated a significantly reduced peripapillary RNFL flow index, retinal flow index, and retinal vessel density in the highly myopic eyes with tessellated fundus with respect to the control eyes [7]. In another study, it was revealed that in the highly myopic eyes with peripapillary intrachoroidal cavitation (PICC), peripapillary, inferotemporal, and superotemporal vessel density was lower than in that without PICC [12].

With OCTA, Fan et al. studied 91 eyes from emmetropia to high myopia, including pathologic myopic eyes with peripheral retinal degeneration, and revealed that superficial and deep vascular density in the macula negatively correlated with AL and the degree of myopia, but positively correlated with ganglion cell complex (GCC) thickness [13]. However, the vascular density had no difference in the optic disc area among the groups, without any correlation with AL, SE, or RNFL thickness [13]. Recently, using OCTA, Al-Sheikh et al. also showed that the density of retinal capillary microvasculature was reduced and the area of flow deficit in choriocapillaris (CC) is increased in the greater myopic eyes [5]. These OCTA studies about vasculature changes in the myopic eyes in literature have been summarized and compared (Table 5).

Our findings agree with these previous descriptions about peripapillary vessel density and go further to illustrate that the vasculature at the nasal quadrant of the retina may be more vulnerable to myopia-related structural change than that at other quadrants. However, the clear mechanism of these graphical correlations in myopia disease development remained unknown. Previous researchers have stated one possible hypothesis that thinning of peripapillary RNFL may affect the vasculature network via autoregulatory mechanisms [1]. Another explanation from the view of myopia pathology is that the elongation of the globe leads to the decreased peripapillary RNFL and vessel density. However, according to our results, the reducing trend of peripapillary RNFL as myopia grows differs from that of peripapillary vessel density at retina quadrants, which is in conflict with the hypothesis. If the change of peripapillary RNFL directly contributes to the vessel distribution or the elongation of the myopic globe contributes to the loss of RNFL and vasculature physically and structurally, the localization of changes of the two should be at similar retina quadrants. Thus, further studies are necessary to explain the disagreement between the patterns of peripapillary RNFL thickness, vessel density, and AL.

The current study had several limitations. First, the cross-sectional design of the study precluded a causative conclusion that AL elongation in myopia induced the thinning of RNFL and vascular density at quadrants in the peripapillary area. Second, the sample size in the present study was relatively small and from the same race within a narrow spectrum of age, mainly in twenties and thirties. Thus, the study is an exploratory and descriptive analysis, and its conclusions may not be applicable to the other races or age spectrums, for example, Caucasian, African, children, or elderly. Third, we did not adjust the confounding factors such as age, which might affect the accuracy of statistical analysis. Fourth, since the Angio Retina program used in this OCTA study divided the peripapillary RNFL and vessel density into four quadrants only (superior, inferior, nasal, and temporal), further researches with more detailed division and new generation of OCTA analyzing program are required to investigate the structural changes in the myopic eyes from the sectional perspective.

OCTA as a novel, high-speed, and noninvasive imaging technique to detect blood flow signals in the retina and the choroid has advantages over the traditional angiography techniques. As shown in Table 4, OCT-based techniques have been widely utilized to study the retinal changes in myopia, and results from OCTA studies agree with those from the OCT. It indicates that OCTA could be used as an efficient replacement of the OCT in the research about RNFL and vasculature in myopia as well as in other fundus diseases. Like all the other imaging techniques, OCTA also has limitations and shortages. First, it requires the patients to keep a stable position and fixation, which makes it impossible for the weak, elderly, or patient with nystagmus to undergo the measurement. Second, the scanning scope of the retina by OCTA is relatively small, from 3 Å–3 mm to 6 Å–6 mm, which limits its usage in detecting the peripheral diseases. Moreover, superficial large blood vessel could form artificial projection in the outer retina which is without blood vessels. Therefore, the development of the OCTA technique in the future should be focused on the following aspects: (1) to increase the imaging speed, in order to lower the time of the patient kept fixated and positioned; (2) to widen the scanning range of the OCTA in the retina and choroid; and (3) to improve the quality of imaging by upgrading more plausible and accurate processing software.

In conclusion, using OCT angiography, we have found that peripapillary RNFL thickness decreased significantly at the superior and inferior quadrants as the myopia increased, while there was a negative correlation between the peripapillary vessel density at the nasal quadrant and AL in the myopic eyes. Measuring changes in RNFL structure and vascular density with OCTA could be a useful method to assess and follow the severity of disease in myopic subjects.

## Figures and Tables

**Table 1:** Demographic and ocular characteristics of participants between the refractive groups.

Measurements	EM (*n* = 17)	MIM (*n* = 27)	MOM (*n* = 33)	HM (*n* = 21)	*P* value
Gender (male : female)	8 : 9	9 : 18	16 : 17	9 : 12	0.671
Laterality (OD : OS)	9 : 8	11 : 16	18 : 15	10 : 11	0.565
Age (years)	23.9 ± 2.7	25.0 ± 3.3	22.6 ± 2.1	23.5 ± 4.9	0.113
MSE (dioptres)	0.2853 ± 0.65	−1.7893 ± 0.74	−4.6379 ± 0.81	−7.5976 ± 1.57	*<0.001*
Axial length (mm)	23.2 ± 0.57	24.2 ± 0.92	25.2 ± 0.84	26.5 ± 0.68	*<0.001*
RNFL thickness (*μ*m)					
Average	109.8 ± 5.2	110.3 ± 6.2	105.5 ± 6.3	101.0 ± 8.9	*<0.001*
S	137.5 ± 10.7	140.0 ± 11.2	131.9 ± 13.9	124.2 ± 14.8	*0.002*
N	81.4 ± 5.8	83.2 ± 8.7	79.4 ± 14.0	76.0 ± 16.5	0.211
I	139.4 ± 10.4	140.3 ± 14.1	132.1 ± 11.3	123.1 ± 14.9	*<0.001*
T	80.4 ± 7.1	77.7 ± 12.0	79.1 ± 11.3	78.3 ± 16.2	0.919
Vessel density (%)					
Average	59.9 ± 3.5	61.3 ± 2.7	59.9 ± 3.9	58.7 ± 3.6	*0.023*
S	59.7 ± 6.2	62.8 ± 3.7	62.4 ± 3.9	59.7 ± 6.3	*0.028*
N	57.8 ± 3.8	57.7 ± 3.7	55.7 ± 4.4	53.9 ± 4.1	*0.016*
I	60.9 ± 3.1	63.0 ± 3.0	61.5 ± 5.4	61.8 ± 3.7	0.206
T	58.5 ± 4.9	61.7 ± 4.0	60.0 ± 5.2	59.3 ± 3.0	*0.015*

Numbers appear as mean ± SD. I: inferior quadrant; MSE: mean spherical equivalent; N: nasal quadrant; RNFL: retinal nerve fibre layer; S: superior quadrant; T: temporal quadrant. *P* values significant at 5% level, Kruskal-Wallis test. Significant values are in italic type.

**Table 2:** Correlation between overall and regional peripapillary RNFL thickness and myopic measurements.

	AL	SE
Average	*−0.450 (<0.001)*	*0.379 (<0.001)*
S	*−0.335 (0.001)*	*0.339 (0.001)*
N	−0.145 (0.154)	*0.209 (0.039)*
I	**−** *0.551 (<0.001)*	*0.379 (<0.001)*
T	−0.007 (0.942)	−0.026 (0.798)

Numbers appear as correlation coefficient (*P* value). Significant values are in italic type. Comparison of correlation coefficients by the Spearman rank correlation. AL: axial length; I: inferior quadrant; N: nasal quadrant; S: superior quadrant; SE: spherical equivalent; T: temporal quadrant.

**Table 3:** Correlation between overall and regional peripapillary vessel density and myopic measurements.

	AL	SE
Average	*−0.234 (0.020)*	0.152 (0.135)
S	−0.121 (0.236)	0.054 (0.600)
N	−0.053 (0.607)	−0.047 (0.646)
I	*−0.392 (<0.001)*	*0.319 (0.001)*
T	−0.117 (0.252)	0.093 (0.360)

Numbers appear as correlation coefficient (*P* value). Significant values are in italic type. Comparison of correlation coefficients by the Spearman rank correlation. AL: axial length; I: inferior quadrant; N: nasal quadrant; S: superior quadrant; SE: spherical equivalent; T: temporal quadrant.

**Table 4:** Comparison of OCT-based RNFL thickness studies in the myopic eyes.

Division	Method	Conclusion	Reference
None	OCTA	Peripapillary RNFL thickness reduced significantly in high myopia compared to mild myopia.	Wang et al. 2016 [1]
12 o'clock	OCT	Peripapillary RNFL thickness was thinner at 1, 7, and 12 o'clock sectors in the highly myopic eyes than in the mild myopic eyes.	Leung et al. 2006 [9]
12 o'clock	Cirrus HD-OCT	RNFL thickness of the 1, 2, 5, 6, and 12 o'clock sectors was significantly thinner in moderate to high myopia than in mild myopia.	Seo et al. 2017 [11]
None	OCT-1	Mean peripapillary RNFL thickness did not vary with myopic SE or axial length for any OCT scan diameter investigated.	Hoh et al. 2006 [14]
12 o'clock	Stratus OCT	The RNFL thickness in high myopia decreased significantly at 1, 5, 6, 7, 8, 9, 10, 11, and 12 o'clock.	Efendieva 2014 [10]
Quadrant/12 o'clock	Cirrus HD-OCT	Average and temporal RNFLs increased significantly as the AL increased.	Choi et al. 2014 [15]
Quadrant	SD-OCT	Global and the temporal RNFL were thicker in the myopia group.	AttaAllah et al. 2017 [16]
Six sectors	SD-OCT	RNFL thickness in children was not affected by myopia.	Goh et al. 2017 [17]

**Table 5:** Comparison of OCTA-based fundus vasculature studies in the myopic eyes.

Division	Method	Conclusion	Reference
None	OCTA	Peripapillary retinal perfusion (flow index and vessel density) was lower in higher myopia than emmetropia.	Wang et al. 2016 [1]
None	OCTA	Peripapillary retinal perfusion (flow index and vessel density) reduced in high myopia with tessellated fundus with respect to the control eyes.	Wang et al. 2016 [7]
Six sectors	OCTA	Peripapillary, IT, and ST vessel density in the highly myopic eyes with PICC was lower than the density in those without.	Chen et al. 2017 [12]
None	OCTA	Macular but not optic disc superficial and deep vascular density reduced as AL increased in the myopic eyes.	Fan et al. 2017 [13]
None	OCTA	Retinal capillary density reduced while CC flow deficit increased in greater myopia.	Al-Sheikh et al. 2017 [5]
None	OCTA	Retinal microvascular network alterations in the highly myopic eyes correlates with axial length elongation.	Yang et al. 2016 [18]
None	OCTA	Retinal microvascular decreased in the high myopia subjects with unchanged retinal microvessel blood flow velocity.	Li et al. 2017 [19]
None	OCTA	SE and AL influence the size of the foveal avascular zone.	Tan et al. 2016 [20]

CC: choriocapillaris; PICC: peripapillary intrachoroidal cavitation.
